# Using the patient activation measure to examine the self-management support needs of a population of UK workers with long-term health conditions

**DOI:** 10.1177/17423953211043492

**Published:** 2021-09-27

**Authors:** Sally Hemming, Fehmidah Munir

**Affiliations:** School of Sport, Exercise and Health Sciences, 152602 Loughborough University, UK

**Keywords:** self-management support, workplace, patient activation, chronic illness, long-term health conditions

## Abstract

**Objectives:**

To examine differences in patient activation and self-management support needs in a population of UK workers with long-term health conditions.

**Methods:**

Demographic, health and activation information were taken from the data of participants with long-term conditions, collected via an online cross-sectional survey of workers. The 13-item British patient activation measure measured workers knowledge, skills and confidence towards self-managing.

**Results:**

Three hundred and seven workers with mental health, musculoskeletal and other conditions completed the patient activation measure. Mental health conditions were most prevalent (36.8%). Workers were higher activated, however workers with mental health conditions were significantly less activated (*p* = 0.006). Differences in activation by condition severity and age were revealed.

**Discussion:**

This study provides insight to the activation of UK workers with long-term conditions. Whilst workers with mental health conditions need more training and education to self-manage, workers are variably activated indicating broader support needs. There is a gap for workplace self-management support. The patient activation measure is used in healthcare to improve people’s self-management and should be considered to be included in the workplace, and could form part of interventions to support workers self-management. More rigorous studies, including the patient activation measure, are needed to identify the best approaches to identifying workers self-management support needs.

## Introduction

Long-term health conditions (LTCs) can negatively affect people’s workability, quality of life and wellbeing.^1^ Adverse effects could be alleviated by people self-managing symptoms and seeking workplace support, but self-managing without support can lead to psychosocial and practical problems, which can eventually lead to deterioration of health and exiting from the labour force.^2,3^ The patient activation measure (PAM) reliably assesses people’s self-efficacy and willingness to self-manage an LTC.^4^ Activation is concerned with people’s confidence in managing their health and care. The PAM is used by UK healthcare practitioners to identify patient’s support needs, by assigning individuals an activation level reflecting their engagement to self-manage.^4^ Yet most studies focus on the PAM from patient, not worker, perspectives and in the United States.^4,5^

There is a greater focus in the UK on supporting people’s LTC self-management.^6^ However, there is no research, to our knowledge, utilising the PAM to examine the LTC self-management support needs of UK workers. Understanding these needs could help align support between healthcare and employers.

This study examined the activation of a population of workers with LTCs by three condition groups: mental health; musculoskeletal (MSK); and other (including neurological, endocrine, metabolic and respiratory). Studies suggest that activation varies by LTC^5^ implying different support needs. Consequently, we hypothesised there would be differences in activation between workers with different LTCs.

## Methods

This study formed part of a larger project.^7^ Actively working, UK workers with an email account were asked to participate in an online survey via their employer, LinkedIn or charity advertisement. Participants consented when registering on the online platform. Demographic and self-reported health data including age, gender, education, industry and main LTC (affecting work the most if more than one) was collected between February 2017 and January 2019. Participants were asked to select a medically diagnosed LTC (requiring ongoing treatment/management over a period of years), category (e.g. MSK) and severity as mild, moderate or severe. Ethical approval was granted by Loughborough University Ethical Committee in 2016.

The 13-item PAM^4^ was licensed and independently scored by Insignia to measure workers knowledge, skills and confidence towards self-managing an LTC (*α* = 0.87). Scale scores (0–100) and levels (1–4, recommended by Judith Hibbard) evaluated workers view of themselves as a self-manager. Higher scores and levels suggest more active self-management and support needs to maintain behaviours (defined in Table 1). Lower scores and levels indicate more passivity in self-managing and support needs to initiate self-managing.

**Table 1.** Demographic and activation characteristics including activation scores and level across the condition groups.

Variable	All	Mental health	MSK	Other	*P* ^a^
	*M* (SD)	*M* (SD)	*M* (SD)	*M* (SD)	
Age	42.7 (11.7)	38.2 (11.4)	45.9 (11.0)	46.2 (11.2)	**0.001**
Variable	All	Mental health	MSK	Other	*P* ^b^
	*N* (%)	*N* (%)	*N* (%)	*N* (%)	
Health condition	307 (100.0)	113 (36.8)	92 (30.0)	102 (33.2)	
Condition severity					**0.001**
Mild	72 (23.5)	15 (20.8)	19 (26.5)	38 (52.8)	
Moderate	185 (59.9)	73 (39.5)	56 (30.3)	55 (29.7)	
Severe	51 (16.6)	25 (49.0)	17 (33.3)	9 (17.6)	
Gender					0.671
Female	234 (76.2)	83 (35.5)	71 (30.3)	80 (34.2)	
Male	73 (23.8)	30 (41.1)	21 (28.8)	22 (30.1)	
Education					0.369
Degree	201 (65.5)	80 (39.8)	59 (29.4)	62 (30.8)	
A level or equivalent	68 (22.1)	18 (26.5)	23 (33.8)	27 (39.7)	
GCSE or below	38 (12.4)	15 (39.5)	10 (26.3)	13 (34.2)	
Industry					0.091
Professional services, banking & finance	95 (30.9)	44 (46.3)	24 (26.3)	27 (28.4)	
Public sector, not for profit	88 (28.7)	26 (29.5)	35 (39.8)	27 (30.7)	
Healthcare	81 (26.4)	25 (30.9)	25 (30.9)	31 (38.3)	
Private sector, for profit	35 (11.4)	14 (40.0)	8 (22.9)	13 (37.1)	
Other, unknown	8 (2.6)	4 (50.0)	.	4 (50.0)	
Activation measure	307 (100.0)	113 (36.8)	92 (29.9)	102 (33.3)	
Variable	All	Mental health	MSK	Other	* P* ^a^
	*M* (SD)	*M* (SD)	*M* (SD)	*M* (SD)	
Activation score	62.6 (14.8)	57.9 (14.1)	62.7 (13.5)	67.6 (15.1)	**0.001**
Range	29.0–91.7	33.0–91.7	35.5–91.7	29.0–91.7	
	All	Mental health	MSK	Other	
Variable	*N* (%)	*N* (%)	*N* (%)	*N* (%)	*P* ^b^
Activation level					
Level 1 – passive	53 (17.3)	30 (26.5)	14 (15.2)	9 (8.82)	**0.001**
Level 2 – becoming aware	48 (15.6)	22 (19.5)	15 (16.3)	11 (10.8)	
Level 3 – taking action	118 (38.4)	43 (38.1)	37 (40.2)	38 (37.3)	
Level 4 – maintaining behaviour	88 (28.7)	18 (15.9)	26 (28.3)	44 (43.1)	

*P* value for ^a^ANOVA and ^b^*x*^2^.

Bold values indicate a significant *p* < .001.

*T*-tests for continuous and chi-square tests for categorical variables explored demographic differences between LTC groups. Between-group differences in activation scores were compared using analysis of variance controlling for gender, age and LTC severity (analysis of covariance) and Bonferroni post-hoc tests. Chi-square tests examined differences in activation levels.

## Results

Three hundred and seven participants with mental health (*n* = 113), MSK (*n* = 92) and other (*n* = 102) conditions completed the PAM. Demographic and activation characteristics are available in Tables 1 and 2. Sixty-seven percent of workers were higher activated at levels 3 and 4. Workers with mental health LTCs were least activated (*M* = 57.9) compared to MSK (*M* = 62.7) and other (*M* = 67.6) conditions. Roughly half of participants at levels 1 (56.6%) and 2 (45.8%) reported a mental health LTC. Workers with mental health conditions were significantly younger (*p* = 0.001) and reported worse condition severity (*p* = 0.001). Further study design details are available elsewhere.^7^

**Table 2.** Analysis of covariance results by condition group for activation.

	Mental health *n* = 113		MSK *n* *=* 92		Other *n* *=* 102		
Source of variance	Observed mean (SD)	Adjusted mean (SE)	Observed mean (SD)	Adjusted mean (SE)	Observed mean (SD)	Adjusted mean (SE)	*P*
Activation score (*n* = 307)	57.9 (14.1)	59.7 (1.35)	62.7 (13.5)	62.2 (1.46)	67.6 (15.1)	66.1 (1.40)	**0.006**

Covariates gender, age and LTC severity.

Bold values indicate a significant *p* < .05.

The study hypothesis was supported. A significant association between LTC and activation was revealed (*F*(2,301) = 5.25, *p* = 0.006) (Table 2). Bonferroni post-hoc tests showed significant differences in activation between workers with mental health and MSK (*p* = 0.002, 95% CI [−7.02, 2.49]) and other LTCs (*p* = 0.001, 95% CI [−10.6, −1.14]). Differences in activation by LTC severity (*F*(1, 301) = 13.36, *p* = 0.001) and age (*F*(1, 301) = 9.60, *p* = 0.002) independent of LTC were revealed.

## Discussion

This study provides new insight to a population of UK workers with LTCs who were largely activated to self-manage. However, a third of participants had low activation levels. Significant differences in activation between workers with mental health and all other LTCs was found. Those with mental health conditions had lower activation levels, mostly scoring in the ‘passive’ or ‘becoming aware’ levels. This feasibly suggests that workers with mental health conditions need more training and education in how to self-manage. Studies do not exist to directly compare workers activation, but the findings are comparable to reports of lower self-efficacy in workers with mental health conditions.^8^ These findings reinforce an occupational need for mental health support.^9^ Nonetheless, between-group differences in activation suggest workers have variable support needs, indicating a broader need for workplace LTC self-management support. Yet workers with LTCs themselves should not be viewed in isolation because employers need the right resources, including manager training, to adequately support workers’ self-management.^7^

While a need for mental health self-management support was indicated and reinforced by condition severity, higher activation does not negate support needs. The overall differences in activation levels suggest that workers with all LTC types need some form of self-management support, whether passive, becoming aware, taking action or maintaining behaviour. Other highlighted factors add to existing evidence that worsening LTCs indicate declining self-management confidence,^10^ and corroborating studies relating older age to better self-management.^11^ Given such complexities, understanding worker’s activation could help employers identify people’s self-management support needs. This study supports and furthers^5^ recommendations that activation could form part of general workplace intervention programmes to support workers’ health, though specifically for workers self-managing LTCs.

The study’s sample size, gender-split and cross-sectional nature limits the findings generalisability, but highlights an important gap for employer’s support. The PAM is routinely used in healthcare settings to improve people’s LTC self-management^4^ and should be considered to be included in workplaces. As workers with other LTCs were more activated, perhaps due to condition variability, time since diagnosis and support, future research deciphering the reasons for differences could help identify support needs and inform interventions to motivate workers’ self-managing different LTCs. Accounting for condition severity, age and time since diagnosis would be insightful.

Employers want workers with LTCs to be well and able to work. To achieve this, work interventions are needed to support workers LTC self-management. Measuring workers activation could help employers (and occupational health) support workers engagement to self-manage LTCs, by identifying support needs and broadening workplace wellbeing offerings. More rigorous studies are needed to identify the best approaches to identifying workers self-management support needs including the practical and economic feasibility of the PAM.
